# Choroidal and Retinal Vascular Findings in Patients with COVID-19 Complicated with Pneumonia: Widefield Imaging

**DOI:** 10.3390/diagnostics13061114

**Published:** 2023-03-15

**Authors:** Rossella D’Aloisio, Maria Ludovica Ruggeri, Giada D’Onofrio, Federico Formenti, Matteo Gironi, Marta Di Nicola, Annamaria Porreca, Lisa Toto, Rodolfo Mastropasqua

**Affiliations:** 1 Ophthalmology Clinic, Department of Medicine and Science of Ageing, D’Annunzio University of Chieti-Pescara, Via dei Vestini 31, 66100 Chieti, Italy; 2 Department of Medical, Oral and Biotechnological Sciences, Laboratory of Biostatistics, D’Annunzio University of Chieti-Pescara, Via dei Vestini 31, 66100 Chieti, Italy

**Keywords:** COVID-19, widefield OCT-A, choroidal vascularity index, vessel density

## Abstract

Purpose: The purpose of this study was to analyze choroidal and retinal vascular alterations of both the macula and midperiphery areas in patients hospitalized for COVID-19 infection complicated with pneumonia within 30 days from discharge. Methods: A total of 46 eyes of 23 subjects with a history of symptomatic COVID-19 infection and recent hospitalization for pneumonia were enrolled in this observational study. Patients had not been previously vaccinated against COVID-19. A group of patients homogenous for age and sex was enrolled as controls. Microvascular retinal and choroidal features of the enrolled patients were studied with widefield optical coherence tomography angiography (OCT-A). Perfusion parameters in terms of the vessel density (VD) of the superficial capillary plexus (SCP) and deep capillary plexus (DCP) and the choroidal vascularity index (CVI) on enhanced depth imaging (EDI) mode OCT scans were analyzed. Results: Our cohort of patients showed a trend of reduction in VD, significantly in the SCP VD of the superior and inferior midperiphery sectors, whereas the CVI did not show significant differences between the cases and controls. Moreover, a positive correlation between CVI and vessel density in the deep capillary plexus in the macular area (VD-DCP-MAC) was found. Conclusion: The systemic disease due to COVID-19 can also involve the retina and choroid with multiple mechanisms: ischemic and inflammatory. Our study showed changes in perfusion occurring in the eyes of patients with a recent hospitalization for COVID-19 complicated with pneumonia and without any possible ocular effect due to the vaccines. There is still the need to better comprise how long COVID-19 actually affects vascular changes in the eye.

## 1. Introduction

COVID-19 is a disease mainly characterized by respiratory system involvement that can lead to acute respiratory distress syndrome. Thrombotic complications are described in patients with a severe form of the disease [1]; however, gastrointestinal, cardiovascular, neurological, dermatological, renal, and ocular manifestations are also reported [2,3].

The angiotensin-converting enzyme (ACE) 2 receptor seems to be the receptor that allows the entry of SARS-CoV-2 in human cells and is also expressed in some ocular structures, such as the retina, ciliary body, and choroid [4].

SARS-CoV-2 was found in tears, conjunctiva, and the retina [5,6,7,8]. Although ocular symptoms in patients with COVID-19 are relatively rare and are referred mainly with dry eye, foreign body sensations, ocular redness, conjunctivitis, and blurry vision [9], some studies have described ocular findings in patients with a severe COVID-19 form, such as retinal hemorrhages, cotton wool spots, and retinal vein occlusions [10,11,12,13].

With the development of retinal multimodal imaging, it has been possible to study retinal and choroidal vasculature structures also in these types of patients in a safe and fast fashion by means of optical coherence tomography (OCT) and optical coherence tomography angiography (OCT-A) scanning. Some retinal alterations have been already reported in COVID-19 patients with a severe form of the disease compared with healthy controls, including retinal vessel enlargement, tortuous vessels, and lower vessel density of the superficial capillary plexus (SCP) and deep capillary plexus (DCP) [10,14,15,16].

Moreover, during the active period of the disease, compared to after a recovery period and healthy controls, authors have detected increased outer plexiform layer thickness, higher peripapillary RNFL, and choroidal changes as well, including a lower choroidal vascularity index and an increased choroidal thickness [17].

Previous studies focused on the perfusion changes of the macula and did not investigate the periphery. The aim of this observational study was to explore and quantify topographical vascular modifications, assessing and correlating perfusion parameters of the macula with those of the midperiphery by means of widefield optical coherence tomography angiography (WF-OCT-A) in such a particular cohort of patients suffering from COVID-19 without any vaccination and hospitalized for interstitial pneumonia. Choroidal changes with the choroidal vascularity index (CVI) have been investigated as well.

## 2. Materials and Methods

### 2.1. Study Participants

In this cross-sectional observational study, 46 eyes of 23 subjects (57% males and 43% females aged between 45 and 60) with a history of symptomatic COVID-19 infection confirmed with a positive test result with real-time reverse transcription–polymerase chain reaction of a nasopharyngeal swab sample and with a history of recent (within 1 month) hospitalization at the Infectious Diseases Clinic, University G. d’Annunzio, SS Annunziata Hospital of Chieti (Italy) were enrolled at the Ophthalmology Clinic of University G. D’Annunzio, Chieti–Pescara, Italy from April 2021 to October 2022. Patient characteristics are shown in Table 1.

A total of 46 eyes of 23 patients homogeneous for age and sex, without COVID-19 vaccination, without history of COVID-19 infection, and with a negative COVID-19 nasopharyngeal swab sample polymerase chain reaction (PCR) result within 7 days from the examination were enrolled as control group.

Exclusion criteria included (1) ocular media opacities (according to the Lens Opacities Classification System III), (2) previous ocular surgeries within 6 months, (3) evidence or history of other ocular diseases, or (4) autoimmune or other uncontrolled systemic diseases affecting the eye.

This study adhered to the tenets of the Declaration of Helsinki and was approved by our Institutional Review Board of the University “G. d’Annunzio” of Chieti–Pescara (Department of Medicine and Science of Ageing), and all patients provided written informed consent for participation in this study.

### 2.2. Procedures

All patients underwent a complete ophthalmic examination which was performed by an expert retinal specialist ophthalmologist, including best corrected visual acuity (BCVA) evaluation (measured using the Early Treatment Diabetic Retinopathy Study (ETDRS) charts), Goldmann applanation tonometry, slit-lamp biomicroscopy, and indirect fundus ophthalmoscopy. In addition, spectral domain (SD)-OCT and OCT-A were performed using Spectralis^®^ HRA+OCT (Heidelberg Engineering; Heidelberg, Germany) and widefield swept-source OCT-A platform PLEX Elite 9000 device (Carl Zeiss Meditec Inc., Dublin, CA, USA), respectively. Biometry for axial length calculation was assessed (IOL Master 700, Carl Zeiss Meditec Inc., Dublin, CA, USA).

### 2.3. Optical Coherence Tomography Analysis

OCT images were obtained using the Spectralis OCT (Heidelberg Engineering, Inc., Heidelberg, Germany). The acquisition protocol for SD OCT included 49 horizontal raster dense linear B-scans centered on the fovea and both horizontal and vertical B-scans centered on the fovea with enhanced depth imaging (EDI) mode, obtaining scans with an axial resolution of 7 microns in tissue.

Images with poor signal strength (<25) were excluded and thus repeated until good-quality scans were obtained.

CVI was measured through the application of a validated algorithm as previously described [18,19,20]: Manual identification of the choroid was performed, defined as the area between the outer border of the RPE and the sclera, and thus known as total choroidal area (TCA). Images were binarized through “Niblack’s Auto Local threshold”, dark pixels were defined as the luminal area (LA), and light pixels were defined as stromal area (SA) [21]. CVI percentage (%) was obtained by dividing LA for TCA.

### 2.4. Optical Coherence Tomography Angiography Analysis

Microvascular retinal and choroidal features of enrolled patients were studied with PLEX Elite 9000 device (Carl Zeiss Meditec Inc., Dublin, CA, USA). The latter uses a swept laser source with a central wavelength of 1050 nm (1000–1100 nm full bandwidth) and operates at 100,000 A-scans per second. It is characterized by a full width at half maximum (FWHM) axial resolution of approximately 5 μm in tissue and a lateral resolution at the retinal surface estimated at approximately 14 μm [22].

In our study, FastTrack motion correction software was used, and poor-quality scans (with either significant motion artifact, incorrect segmentation, or signal strength < 8) were excluded and thus repeated until good-quality scans (defined as scans with a signal strength ≥ 8) were achieved.

For each eye, 12 × 12 mm volume scans centered on the fovea were performed. All vascular retinal layers were identified and segmented in the SCP and DCP [23]. The projection-resolved algorithm was used to eliminate projection artifacts. This algorithm retains flow signals from blood vessels and suppresses projected flow signals in deeper layers [24].

**Figure 1:**
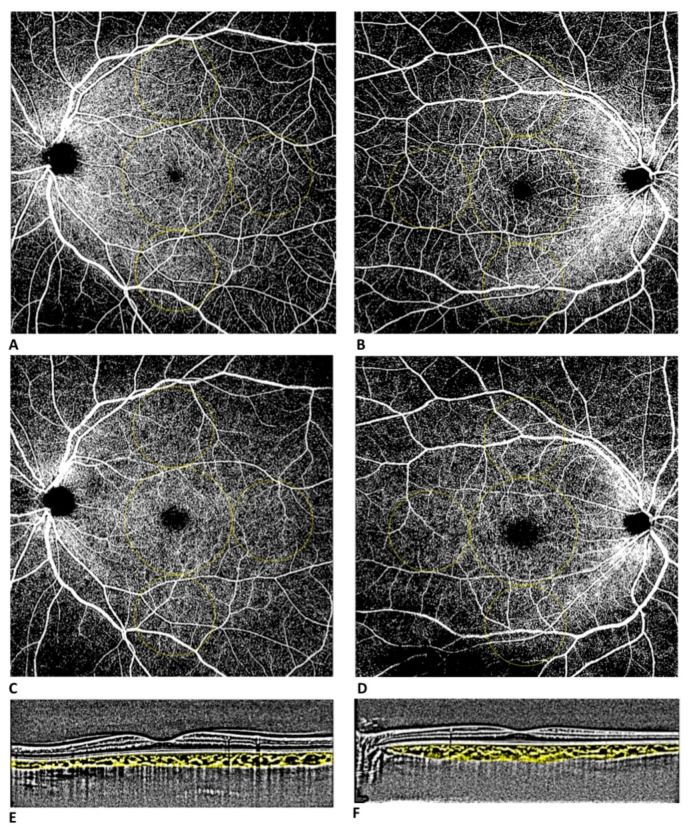
Widefield OCT-A assessment (12 × 12 mm scan) of superficial (**A**) and deep (**C**) capillary plexus of a healthy control patient. Widefield OCT-A assessment of superficial (**B**) and deep (**D**) capillary plexus of a patient with a history of symptomatic COVID-19 infection. Vessel densities were defined in two different regions: macular region (central circle with a diameter of 4 mm) and midperiphery region (3 circles with diameter of 3 mm tangential to the central circle). Dense linear B-scans centered on the fovea with enhanced depth imaging (EDI) of a healthy control patient (**E**) and of a patient with a history of symptomatic COVID-19 infection (**F**).

Images were then analyzed by two retina specialists for segmentation accuracy and then exported as JPEG files in order to be analyzed with ImageJ software version 1.52° (National Institutes of Health, Bethesda, MD, USA; available at http://rsb.info.nih.gov/jj/index.html accessed on 1 October 2022). Vessel density (VD) was defined as the percentage of the area occupied by vessels in a circular region of interest (ROI) and was calculated in the macular area (4 mm in diameter located in the center of the foveal avascular zone; VD MAC) and in 3 circles of 3 mm in midperiphery (VD I, T, S; Figure 1). It was calculated in the SCP and DCP.

### 2.5. Statistical Analysis

Descriptive statistics: Median and 1st and 3rd quartiles were calculated to characterize the study variables. Normal distribution was verified with the Jarque Bera test. The Mann–Whitney U test was applied to investigate whether there were significant differences between cases and controls for all visual parameters. Spearman’s rho correlation coefficient with listwise deletion was calculated to assess the relationship of the continuous parameters in cases and controls. All statistical tests were 2-sided with a significance level set at *p* ≤ 0.05. Statistical analysis was performed using the R environment for statistical computing and graphics version 3.6 (R Foundation for Statistical Computing, Vienna, Austria; https://www.R-project.org/).

## 3. Results

A total of 46 eyes of 23 subjects with symptomatic COVID-19 infection complicated with pneumonia and not vaccinated were enrolled. The 46 eyes of 23 age-matched patients without a history of COVID-19 infection within 7 days from enrollment were considered as the controls in the analysis.

**Figure 2:**
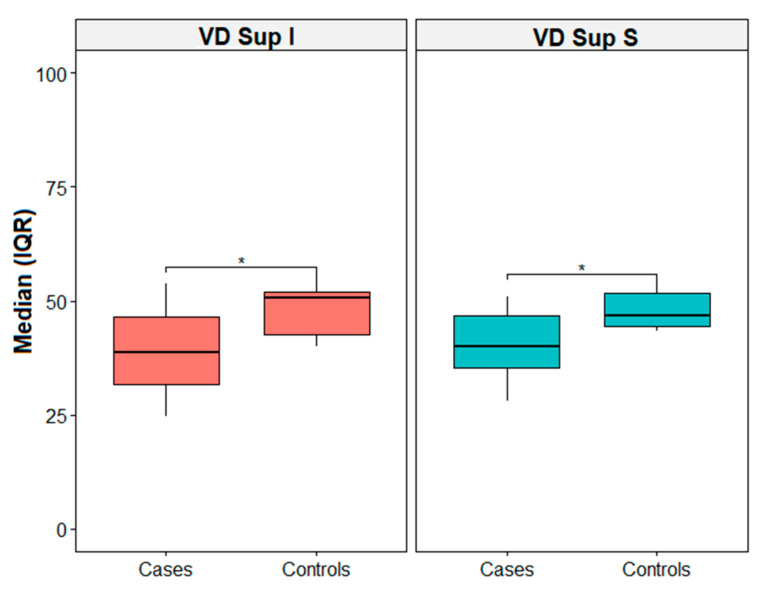
Box plot for VD Sup I and VD Sup S. * = *p*-value < 0.05. IQR = interquartile range.

**Figure 3:**
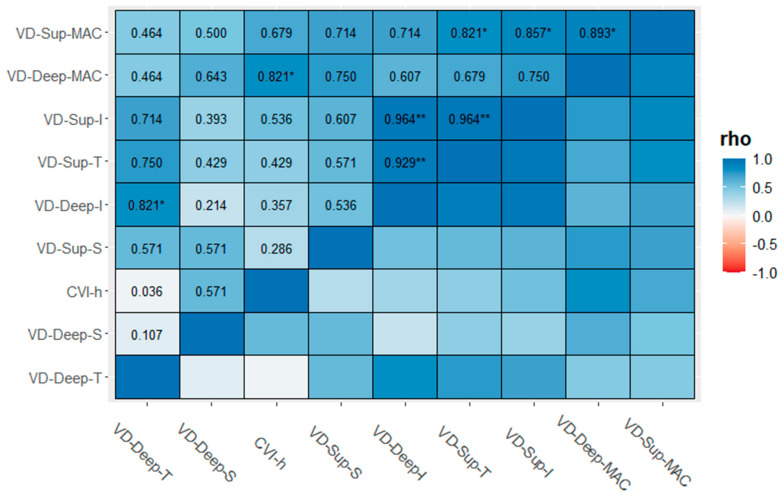
Correlation matrix (CM) for cases computed using the Spearman and listwise deletion methods. Significance level codes: ** *p* < 0.01, and * *p* < 0.05.

Table 2 shows descriptive statistics reported as the median, first (q1), and third (q3) quartile for the cases and controls. Indeed, differences between the groups for the visual parameters were evaluated with the Mann–Whitney U test. Thus, there was a statistically median difference between the cases and controls for VD-SCP-S (46.7 [44.4; 51.8] vs. 40.1 [35.4; 46.7], *p* = 0.039) and VD-SCP-I (50.6 [42.6; 52.1] vs. 38.7 [31.7; 46.6], *p* = 0.026) (Figure 2). Figure 3 shows the correlation matrix using the Spearman rho correlation coefficient for the cases. The data revealed a strong statistically significant positive correlation between VD-SCP-MAC and VD-DCP-MAC (rho = 0.893), and it is important to highlight that there exists a strong positive statistically significant correlation between CVI and VD-DCP-MAC (rho = 0.821).

## 4. Discussion

The possible involvement of the eye during COVID-19 infection has been widely described. As Li et al. recently observed, the presence itself of the ACE2 and CD147 receptors in the human eye indicates their potential role in inducing the ocular manifestation of COVID-19 infection [25].

Several retinal alterations have been described in patients with SARS-CoV2 infection, including retinal vascular changes [13,26,27]. Although the literature about these is limited, some studies described a reduction of VD in diseased patients if compared to healthy controls. Turker et al. found a lower VD in the foveal and parafoveal area of the SCP and in all macular quadrants in the DCP in patients who were hospitalized for COVID-19 disease after 1 week from discharge [28]. Abrishami et al. also described a significantly reduced macular SCP VD and DCP VD two weeks after recovery [14].

The aim of our study was to quantify choroidal and retinal vascular modifications of both the macula and midperiphery areas in patients hospitalized for COVID-19 complicated with pneumonia within 30 days from discharge.

To our knowledge, this is the first study exploring topographically retina and choroid vasculature changes in such a cohort of patients with COVID-19 complicated with pneumonia without COVID-19 vaccination by means of the widefield imaging modality.

Our choice to include nonvaccinated patients was due to the reported ocular findings in patients who underwent COVID-19 vaccination. In fact, although vaccination and subsequent boost administrations remain the main factors influencing and thus reducing the spread of the COVID-19 infection, different adverse outcomes have been reported in the literature. Previous papers have demonstrated a direct or indirect connection between COVID-19 infection and ocular complications [29,30].

A recent one-year retrospective review has reported a detailed description of all findings about COVID-19 vaccination and ophthalmic manifestations [31]. Among them, choroiditis has been reported in two different reports [32,33]. Acute retinal necrosis due to varicella zoster virus (VZV) following the first dose of the AZD1222 vaccine has been described by Mishra et al. as well [34]. In their review of 12 reports focused on retinal manifestation occurring after vaccination, paracentral acute midmaculopathy (PAMM) was described by a limited number of reports [35,36]; however, the most commonly reported adverse effect was found to be AMN [37,38,39]. Previous studies have reported the underlying pathological mechanisms of AMN to be ischemia of the DCP in the inner nerve fiber layer [40,41].

Thus, the strength of our cohort of patients was that the analyzed final outcomes were not influenced by the possible effect of a previous COVID-19 vaccination.

Moreover, all our patients, who had a period of hospitalization for pneumonia, experienced ocular surface symptoms, such as dry eye discomfort, burning, redness, and blurred vision. Such symptoms have yet to be reported both in the general population due to the increased use of face masks and as ocular manifestations of COVID-19 infection [42,43,44]. We hypothesized these findings in our cohort of patients to be attributable to the long-term use of respiratory devices in the intensive care unit.

In our study mainly focusing on the occurring vascular changes, we observed a trend of a reduction in VD with a significant decrease in the SCP in the superior and inferior quadrants of midperiphery compared to controls. Some retinal diseases, such as diabetic retinopathy, retinal vein occlusions, vasculitis, etc., have an impact not only on the macula but also on the midperiphery in terms of ischemia, and some differences between the center and the midperiphery can be assessed. We wanted to observe if some midperipheral changes were present in these patients with COVID-19 and if there was some interesting correlation between macular perfusion and peripheral perfusion.

Other studies focused their attention on the choroid and its modifications in these types of patients using the novel marker CVI, a robust representative of the choroid in both its vascular and stromal factor, giving new additional structural information and enriching the knowledge of COVID-19-related physiopathological ocular conditions. Abrishami et al. calculated the CVI, finding an increased CVI in patients with COVID-19 one month after recovery that returned to baseline values (at recovery) after three months [45].

Others showed a decreased CVI during the COVID-19 disease with a reversible fashion some months after discharge, suggesting reversible and multiple mechanisms (ischemic and inflammatory) of COVID-19 on ocular perfusion [17,46].

Conversely, in our study, the CVI did not show significant differences between the cases and controls, while a strong positive correlation between the CVI and VD-DCP-MAC was found. Given that one of the most important advantages of the CVI is that it is not influenced by a systemic and local factor (age, axial length, or systemic or ocular pressure), it remains a good and specific indicator of choroid vascularization [47].

Our study underlined that, although there was an absence of changes in the CVI, the VD measured in the superficial and deep plexus showed alterations compared to healthy subjects, thus demonstrating, on one hand, the presence of changes in perfusion in the eyes of patients with a recent hospitalization for COVID-19 complicated with pneumonia and without any possible ocular effect due to vaccines and, on the other hand, reinforces the role of the VD parameter as a good indicator of retinal vascular changes occurring in the eye. The systemic disease due to COVID-19 can involve also the retina and choroid with multiple mechanisms, ischemic and inflammatory, since some alterations seem to be reversible, as previously described.

As Byram observed, these vascular alterations may be related both to a direct host–virus interaction or an underlying autoimmune process inducing vasculitis or inflammation [17]. For this reason, our observational study focused on this type of subject without proper vaccination for COVID-19 to avoid some COVID-19 vaccine-related retinal changes. Although some of these could be transient and reversible, we decided to avoid this possible bias. Moreover, the controls have been properly selected without any relevant systemic diseases (only well-controlled diseases not affecting the eye) and without recovery from COVID-19 and were obviously not vaccinated, thus permitting a clear analysis without possible bias.

The strength of our study was that it explored vascular changes in COVID-19 patients with a previous hospitalization for COVID-19 by studying, through widefield OCT-A examination, a wider area, including both the central macular area and the retinal mid periphery.

However, one of the shortcomings of this study was that other functional parameters (contrast sensitivity, visual field) were not investigated. Moreover, a longer follow-up period may be interesting to see how perfusion changes behave over a long time and whether they are reversible.

Further studies are needed to better comprise how long COVID-19 actually affects vascular changes in the eye. COVID-19 and retinal vascular changes are known in the literature and are still questioned if they are reversible or not. This work could add a new understanding of the pathogenesis of COVID-19 to depict a small, selected cohort of patients complicated with pneumonia similar to our sample study to observe if some vascular findings visible on OCTA could predict the prognosis of the patient.

## Figures and Tables

**Table 1:** Demographic characteristics, ophthalmic features, and therapy of enrolled patients.

Patients Characteristics	Cases	Controls
**Demographic Feature**		
Age (years)	50.2 ± 7.8	54.8 ± 9.4
Gender, male/female (%)	57.1/43.2	58.1/42.3
Well-controlled diseases not affecting the eye (%)		
Systemic hypertension	14.0	0.0
Obesity	14.0	0.0
Hypercholesterolemia	28.0	14.0
Hypothyroidism	0.0	14.0
Complete cycle of Vaccination (%)	0.0	0.0
**Ophtalmic features**		
BCVA- ETDRS (letters)	52 ± 2	52 ± 1
Axial length (mm)	23.2 ± 0.9	23.5 ± 0.5
Intraocular pressure (mmHg)	15.7 ± 3.2	14.9 ± 2.5
**Therapy**		
Period of hospitalization (days)	19.0 ± 2.0	/
CPAP (Continuous positive air pressure) therapy (%, days)	58.0	/
	17.0 ± 5.0	/
Ventimask O_2_ therapy (%, days)	80.0	/
	11.0 ± 4.0	/
Dexamethasone therapy (%)	71.0	/
Antiviral therapy (%)	57.0	/

**Table 2:** Summary descriptive table presented as median [q1 = first quartile; q3 = third quartile] for cases and controls. *p*-value derived from Mann–Whitney U test.

Variables	Controls	Cases	*p*-Value
	*n =* 46	*n =* 46	
VD-SCP-MAC	37.1 [33.3; 47.0]	36.7 [31.8; 40.5]	0.509
VD-SCP-S	46.7 [44.4; 51.8]	40.1 [35.4; 46.7]	0.039
VD-SCP-I	50.6 [42.6; 52.1]	38.7 [31.7; 46.6]	0.026
VD-SCP-T	26.6 [25.8; 29.2]	17.8 [16.1; 27.7]	0.161
VD-DCP-MAC	35.2 [32.3; 45.4]	39.0 [33.5; 41.0]	0.934
VD-DCP-S	40.3 [37.9; 42.6]	37.4 [34.7; 44.5]	0.869
VD-DCP-I	43.1 [36.6; 44.5]	33.9 [30.4; 40.4]	0.216
VD-DCP-T	29.6 [22.3; 33.8]	24.2 [19.2; 35.6]	0.934
CVI	0.66 [0.65; 0.68]	0.66 [0.64; 0.68]	0.807

VD, vessel density; SCP, superficial capillary plexus; DCP, deep capillary plexus; MAC, macular; I, inferior; T, temporal; S, superior; CVI, choroidal vascularity index. *n* = 46 eyes of 23 subjects in both groups.

## Data Availability

All data generated or analyzed during this study are included in this article. Further inquiries can be directed to the corresponding author.
